# Comparison of left atrial function in healthy individuals versus patients with non-ST-segment elevation myocardial infarction using two-dimensional speckle tracking echocardiography

**DOI:** 10.5830/CVJA-2013-011

**Published:** 2013-06

**Authors:** Zhu Jing, Chen Jianchang, Xu Weiting, Gao Lan, Farhan Shaikh, Wu Yanni

**Affiliations:** Department of Cardiology, Second Affiliated Hospital of Soochow University, Suzhou, China; Department of Cardiology, Second Affiliated Hospital of Soochow University, Suzhou, China; Department of Cardiology, Second Affiliated Hospital of Soochow University, Suzhou, China; Department of Cardiology, Second Affiliated Hospital of Soochow University, Suzhou, China; Department of Cardiology, Second Affiliated Hospital of Soochow University, Suzhou, China; Department of Cardiology, Second Affiliated Hospital of Soochow University, Suzhou, China

**Keywords:** two-dimensional speckle tracking echocardiography, strain rate, non-ST-segment elevation myocardial infarction, left atrial function

## Abstract

**Abstract:**

Left atrial (LA) function has been associated with adverse outcomes in patients after acute myocardial infarction. The purpose of the current study was to evaluate LA function in patients with non-ST-segment elevation myocardial infarction (NSTEMI) by two-dimensional speckle tracking echocardiography (2D STE). Fifty-one patients with NSTEMI and 40 age-matched normal control individuals were enrolled in this study. Conventional echocardiographic parameters and global longitudinal strain rate (GLSR) were measured at left ventricular (LV) and LA segments. Compared with healthy subjects, patients with NSTEMI had significantly increased LA volumes but significantly decreased LA emptying fraction and GLSR. LA-GLSR had significant correlations with the 2D Doppler echocardiographic parameters of LA function. In particular, global LA peak negative strain rate during early ventricular diastole (LA-GLSRe) was significantly correlated with both LA 2D Doppler echocardiographic parameters and LV contractile function. This could be suggested as a better indicator to evaluate LA function as a preferred parameter of STE.

## Abstract

According to an authoritative survey, more than one million people die each year from coronary artery disease in China. Recently, impaired left atrial (LA) function and its detrimental effect on coronary artery disease has caused wide concern,^1^ Left atrial function is one of the most important clinical parameters of two-dimensional speckle tracking echocardiography (2D STE), which is an innovative tool for more comprehensive and reliable echocardiographic evaluation of myocardial function.^2^

Compared with Doppler and 2D echocardiography, 2D STE has the advantages of angle independence, and is also less affected by reverberations, side lobes or drop-out artifacts. While this novel echocardiographic method has been frequently used to assess LV function,^3^ it has more recently been used to evaluate atrial function in normal subjects and in conditions with atrial dysfunction.^4^,^5^

The aims of this study were to examine left atrial function using 2D STE in patients with non-ST-segment elevation myocardial infarction (NSTEMI) compared to healthy subjects and to define the feasibility of speckle tracking-based strain rate (SR) imaging for the evaluation of LA dysfunction after acute myocardial ischaemia.

## Methods

Fifty-one patients (43 males and eight females; mean age 62.9 ± 11.1 years) were treated by percutaneous coronary intervention (PCI) for NSTEMI and were included in the study from December 2009 to November 2010, while 40 age-matched healthy subjects (35 males and five females; mean age 60.1 ± 9.8 years) with normal treadmill exercise stress echocardiography and no coronary risk factors were enrolled as a control group.

Patients with atrial fibrillation or flutter, valvular heart disease (of mild or greater severity), and poor left atrial images were excluded. The study protocol was approved by the Ethics Committee of the Second Affiliated Hospital of Soochow University and a written informed consent was obtained from each participant.

Conventional 2D and Doppler echocardiography studies were performed using the Vivid7 Dimension ultrasound system (GE, USA) equipped with a 3S phased-array transducer (frequency range of 1.7–3.4 MHz). Echocardiographies of patients were performed 2.8 ± 0.6 days after NSTEMI. Cardiac dimensions were measured in accordance with recommendations of the American Society of Echocardiography.

M-mode echocardiography was used to measure LV end-diastolic and end-systolic diameters. LV ejection fraction (LVEF) was calculated from apical four- and two-chamber views, using the modified Simpson’s rule. LA volumes were measured using the area–length method from apical four- and two-chamber views, according to the guidelines of the American Society of Echocardiography.^6^

Left atrial maximum volume (LAV_max_) was measured at the end of LV systole, just before the opening of the mitral valve, LA minimum volume (LAV_min_) was measured at the end of LV diastole, right after the closure of the mitral valve, and LA pre-atrial volume (LAV_p_) was obtained from the diastolic frame before initial mitral valve re-opening elicited by atrial contraction. LA reservoir function was assessed using LA total EF = (LAV_max_ – LAV_min_)/LAV_max_, LA conduit function was assessed using LA passive emptying fraction (LAPEF) = (LAV_max_ – LAV_p_)/LAV_max_, and LA booster pump function was assessed using LA active emptying fraction (LAAEF) = (LAV_p_ – LAV_min_)/LAV_p_.

For 2D STE analysis, we obtained 2D gray-scale harmonic images in three apical planes (long axis of LV, four- and two-chamber). Three consecutive heart cycles were recorded and averaged. The frame rate was set between 60 and 90 frames per second.^7^ Echocardiograms were digitally stored and later analysed off-line using acoustic-tracking software (Echo-Pac version 7.0, GE Vingmed).^8^ A 16-segment LV model was obtained from the four- and two-chamber, and long-axis recordings.^9^

**Fig. 1.**
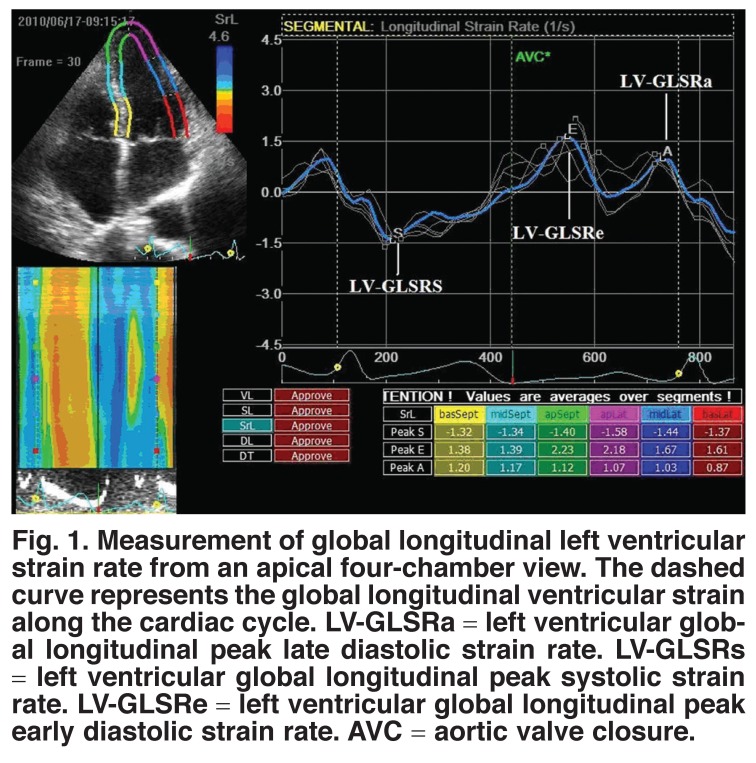
Measurement of global longitudinal left ventricular strain rate from an apical four-chamber view. The dashed curve represents the global longitudinal ventricular strain along the cardiac cycle. LV-GLSRa = left ventricular global longitudinal peak late diastolic strain rate. LV-GLSRs = left ventricular global longitudinal peak systolic strain rate. LV-GLSRe = left ventricular global longitudinal peak early diastolic strain rate. AVC = aortic valve closure.

Two-dimensional strain software identified the endocardial border, and after tracing myocardial motion, was automatically tracked in each imaging view. Strain rate measurements from 16 segments were averaged to assess a LV global longitudinal parameter based on peak systole (LV-GLSRs), early diastole (LV-GLSRe), and late diastole (LV-GLSRa) (Fig. 1).

**Fig. 2.**
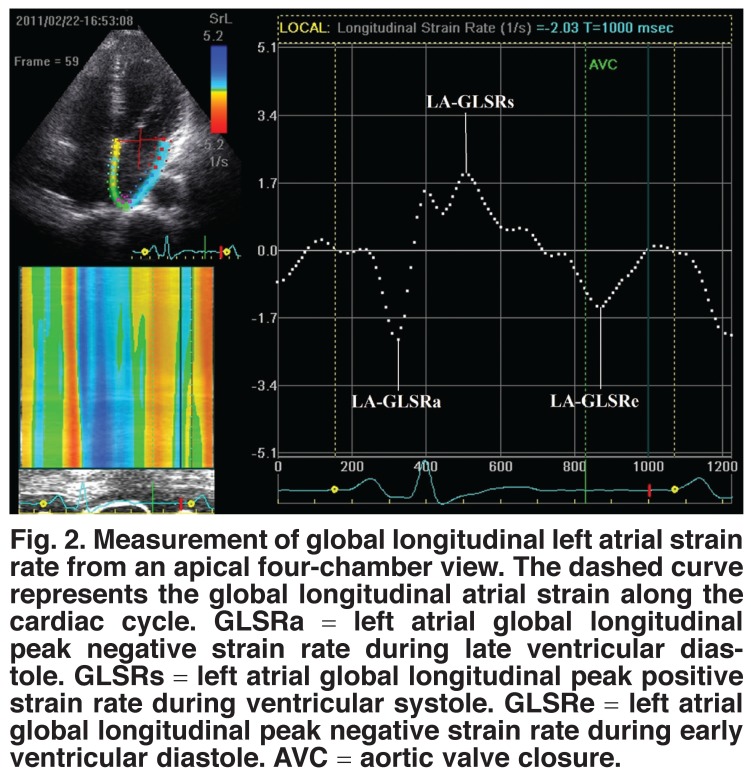
Measurement of global longitudinal left atrial strain rate from an apical four-chamber view. The dashed curve represents the global longitudinal atrial strain along the cardiac cycle. GLSRa = left atrial global longitudinal peak negative strain rate during late ventricular diastole. GLSRs = left atrial global longitudinal peak positive strain rate during ventricular systole. GLSRe = left atrial global longitudinal peak negative strain rate during early ventricular diastole. AVC = aortic valve closure.

The LA myocardium was divided into six equidistant regions from apical four- and two-chamber views, while only three were analysed in the apical long-axis view because the remaining three in this view are part of the aortic valve and ascending aorta and not LA myocardium. The software generates strain rate curves for each atrial segment. Global strain and strain rate were also calculated by averaging values from 15 atrial segments. Lastly, we can get global LA peak positive strain rate during ventricular systole (LA-GLSRs), global LA peak negative strain rate during early ventricular diastole (LA-GLSRe) and global LA peak negative strain rate during late ventricular diastole (LA-GLSRa) (Fig. 2).

To assess inter- and intra-observer variabilities, variabilities in the measurements of LA-GLSRs, LA-GLSRe, LA-GLSRa, LV-GLSRs, LV-GLSRe and LV-GLSRa were evaluated in 20 subjects selected randomly. To assess the inter-observer variability, selected images were analysed by a second observer blinded to the values obtained by the first observer. To assess the intra-observer variability, selected images were analysed at a different time by an observer blinded to the results of the previous measurements.^10^

## Statistical analysis

Data analysis was carried out using the statistical software package (SPSS, Rel 13.0, Chicago: SPSS Inc.). Continuous data were presented as mean ± SD. Differences between the NSTEMI and control groups were assessed by unpaired Student’s *t*-test. Categorical parameters are presented as numbers (%), and were analysed using chi-square tests or Fisher’s exact tests, as appropriate. For assessment of intra- and interobserver variabilities, the Bland-Altman method was used.^11^ The correlation between two variables was assessed using Spearman’s rank correlation coefficient. A two-tailed *p*-value < 0.05 was considered significant for statistical inference.

## Results

The main clinical features and 2D Doppler echocardiography data of the controls and NSTEMI patients are summarised in Tables 1 and 2, respectively. There were significant differences in clinical features, such as hypertension, diabetes and hyperlipidaemia between patients and healthy subjects. Patients with NSTEMI had significantly increased LAV_max_ (60.38 ± 17.64 vs 45.33 ± 14.50 ml, *p* = 0.001), LAV_min_ (25.56 ± 12.59 vs 16.18 ± 8.93 ml, *p* = 0.001), and LAV_p_ (43.80 ± 16.59 vs 27.32 ± 10.74 ml, *p* = 0.001), but significantly lower in LAPEF (28.96 ± 11.62 vs 39.89 ± 13.65%, *p* = 0.001), LA total EF (59.06 ± 13.44 vs 65.53 ± 10.20%, *p* = 0.013) and LVEF (58.08 ± 10.01 vs 65.18 ± 5.22%, *p* = 0.001).

**Table 1:** Clinical Features Of Patients With Nstemi And The Controls

	*Controls (*n* = 40)*	*NSTEMI (*n* = 51)*	p*-value*
Age (years) (mean ± SD)	60.1 ± 9.8	62.9 ± 11.1	0.272
Male, *n* (%)	35 (87.5)	43 (84.3)	0.238
Female, *n* (%)	5 (12.5)	8 (15.7)	0.179
Height (cm)	167.06 ± 6.97	166.67 ± 7.30	0.546
Weight (kg)	61.56 ± 10.16	62.31 ± 9.70	0.626
Smoking	23	41	0.057
Body mass index (kg/m^2^)	57.5	80.4	0.087
Hypertension (%)	1.69 ± 0.16	1.77 ± 0.15	0.001
Diabetes mellitus, *n* (%)	0	28 (54.9)**	0.001
Hyperlipidaemia, *n* (%)	0	12 (23.5)**	0.001
Occluded coronary artery, n (%)	0	26 (51.0)**	–
RCA, *n* (%)	–	2 (3.9)	–
LAD, *n* (%)	–	11 (21.6)	–
LCX, *n* (%)	–	8 (15.7)	–

RCA = right coronary artery, LAD = left anterior descending artery, LCX = left circumflex coronary artery. **p < 0.01.

**Table 2:** Conventional 2D Doppler Echocardiographic Parameters In Patients With Nstemi And The Controls

	*Controls (n = 40)*	*NSTEMI (n = 51)*	p*-value*
LAV_max_ (ml)	45.33 ± 14.50	60.38 ± 17.64	0.001
LAV_min_ (ml)	16.18 ± 8.93	25.56 ± 12.59	0.001
LAV_p_ (ml)	27.32 ± 10.74	43.80 ± 16.59	0.001
LAPEF (%)	39.89 ± 13.65	28.96 ± 11.62	0.001
LAAEF (%)	42.74 ± 11.25	43.89 ± 11.67	0.637
LA total EF (%)	65.53 ± 10.20	59.06 ± 13.44	0.013
LVEF (%)	65.18 ± 5.22	58.08 ± 10.01	0.001

Date are expressed as mean ± SD.

The SR imaging of LA and LV was acceptable in all 40 healthy subjects, whereas four had one inadequately traced segment. The SR imaging of LA and LV was acceptable in 51 patients, whereas five had one inadequately traced segment. Twenty healthy subjects and 20 patients with NSTEMI were randomly selected for the assessment of intra- and inter-observer variabilities in the measurements of LA-GLSRs, LA-GLSRe, LA-GLSRa, LV-GLSRs, LV-GLSRe and LV-GLSRa, respectively.

**Fig. 3.**
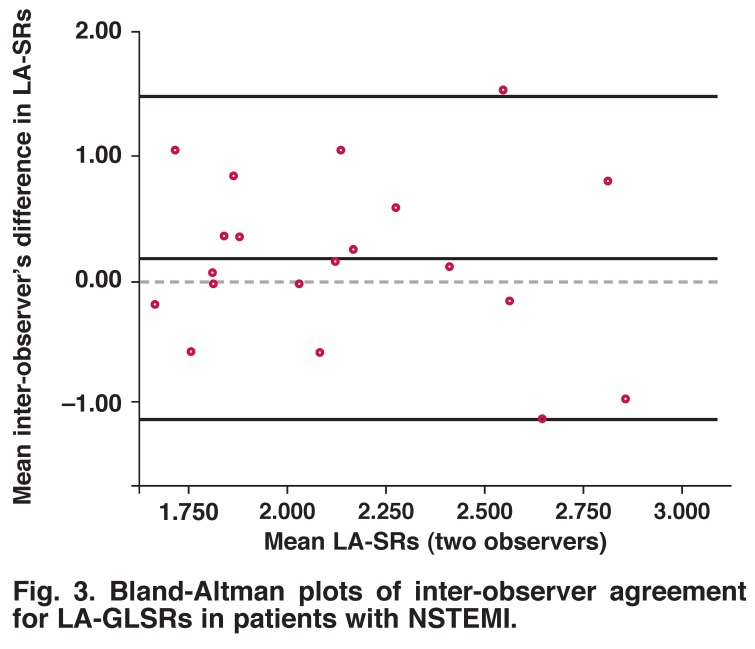
Bland-Altman plots of inter-observer agreement for LA-GLSRs in patients with NSTEMI.

**Fig. 4.**
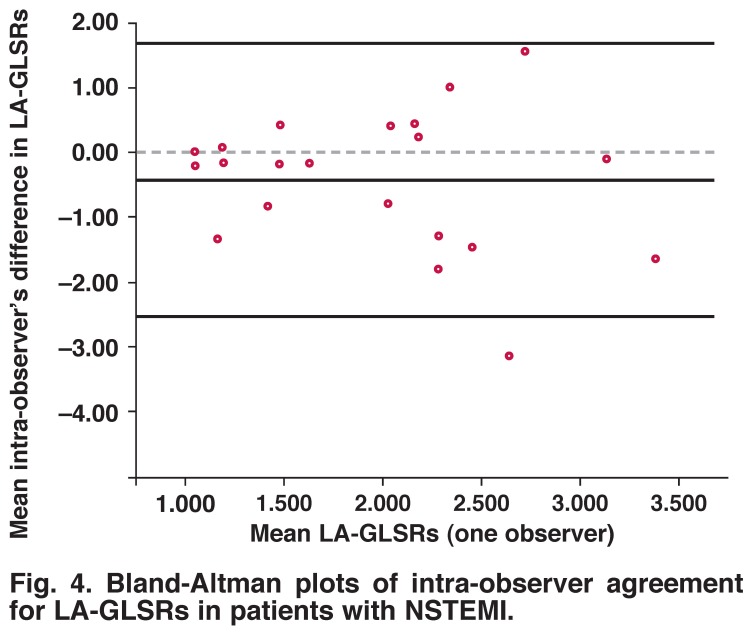
Bland-Altman plots of intra-observer agreement for LA-GLSRs in patients with NSTEMI.

Bland-Altman analysis of these parameters showed no evidence of any systematic difference regarding inter- and intraobserver variabilities. Table 3, and Figs 3 and 4 show the mean difference and confidence intervals of inter- and intra-observer variabilities.

**Table 3:** Reproducibility Of LA And LV Global Strain Rate

	*Controls*		*NSTEMI*	
	*Intra-observer*	*Inter-observer*	*Intra-observer*	*Inter-observer*
LA-GLSRs	0.94 (0.87–0.98)	0.95 (0.88–0.98)	0.95 (0.87–0.98)	0.98 (0.89–0.99)
LA-GLSRe	0.95 (0.88–0.98)	0.97 (0.91–0.99)	0.94 (0.87–0.98)	0.98 (0.90–0.99)
LA-GLSRa	0.94 (0.87–0.98)	0.96 (0.89–0.98)	0.94 (0.87–0.98)	0.93 (0.87–0.98)
LV-GLSRs	0.94 (0.87–0.98)	0.94 (0.85–0.97)	0.82 (0.76–0.96)	0.85 (0.67–0.94)
LV-GLSRe	0.95 (0.88–0.98)	0.94 (0.86–0.97)	0.93 (0.84–0.97)	0.92 (0.81–0.95)
LV-GLSRa	0.93 (0.84–0.97)	0.95 (0.88–0.97)	0.86 (0.80–0.98)	0.93 (0.85–0.97)

LA-GLSRs = LA global longitudinal peak positive strain rate during ventricular systole, LA-GLSRe = LA global longitudinal peak negative strain rate during early ventricular diastole, LA-GLSRa = LA global longitudinal and peak negative strain rate during late ventricular diastole, LV-GLSRs =LV global longitudinal peak systolic strain rate, LV-GLSRe = LV global longitudinal early diastolic strain rate, LV-GLSRa = LV global longitudinal late diastolic strain rate. Date are expressed as mean ± SD.

Tables 4 lists the SR imaging echocardiographic variables of the normal and NSTEMI groups. Compared with the controls, patients with NSTEMI had significantly decreased LA-GLSRs (*p* = 0.001), LA-GLSRe (*p* = 0.001), LV-GLSRs (*p* = 0.004), and LV-GLSRe (*p* = 0.001).

**Table 4:** 2D STE Parameters In Patients With Nstemi And The Controls

	*Controls (n =36)*	*NSTEMI (n = 46)*	*p-value*
LA-GLSRs	1.93 ± 0.48	1.59 ± 0.58	0.001
LA-GLSRe	–2.03 ± 0.70	–1.21 ± 0.52	0.001
LA-GLSRa	–2.25 ± 0.67	–1.90 ± 0.77	0.061
LV-GLSRs	–0.92 ± 0.19	–0.80 ± 0.22	0.004
LV-GLSRe	1.17 ± 0.38	0.78 ± 0.27	0.001
LV-GLSRa	0.71 ± 0.22	0.75 ± 0.21	0.062

LA-GLSRs = LA global longitudinal peak positive strain rate during ventricular systole, LA-GLSRe = LA global longitudinal peak negative strain rate during early ventricular diastole, LA-GLSRa= LA global longitudinal and peak negative strain rate during late ventricular diastole, LV-GLSRs = LV global longitudinal peak systolic strain rate, LV-GLSRe = LV global longitudinal early diastolic strain rate, LV-GLSRa = LV global longitudinal late diastolic strain rate. Date are expressed as mean ± SD.

Correlations of LA-GLSRs, LA-GLSRe, LA-GLSRa, LV-GLSRs, LV-GLSRe and LV-GLSRa with parameters of LA volume and function in NSTEMI patients were performed (Table 5). LA-GLSRs showed modest correlations with parameters of LA volume and function, including LAV_max_ (*r* = –0.610, *p* < 0.01), LAV_min_ (*r* = –0.668, *p* < 0.01), LAV_p_ (*r* = –0.638, *p* < 0.01), LAPEF (*r* = 0.376, *p* < 0.01), LAAEF (*r* = –0.303, *p* < 0.05), LA total EF (*r* = –0.412, *p* < 0.05) and LVEF (*r* = –0.334, *p* < 0.05).

**Table 5:** Correlation Of Global LA/LV Strain Rate Parameters With La Volume And Function Parameters In Patients With Nstemi

*Correlation*	*LAV_max_ (ml)*	*LAV_min_ (ml)*	*LAV_p_ (ml)*	*LAPEF (%)*	*LAAEF (%)*	*LA total EF (%)*	*LVEF (%)*
LA-GLSRs	–0.610**	–0.668**	–0.638**	0.376**	–0.303*	–0.412*	–0.334*
LA-GLSRe	0.586**	0.530**	0.564**	–0.270	0.340*	0.256*	–0.477**
LA-GLSRa	0.604**	0.615**	0.590**	–0.298*	0.262	0.347	0.339
LV-GLSRs	–0.136	–0.165	–0.103	0.089	0.072	0.102	0.361*
LV-GLSRe	–0.062	–0.014	–0.022	–0.042	–0.030	–0.033	–0.414**
LV-GLSRa	0.162	0.203	0.199	–0.102	–0.067	–0.134	–0.405**

LA-GLSRs = LA global longitudinal peak positive strain rate during ventricular systole, LA-GLSRe = LA global longitudinal peak negative strain rate during early ventricular diastole, LA-GLSRa= LA global longitudinal and peak negative strain rate during late ventricular diastole, LV-GLSRs = LV global longitudinal peak systolic strain rate, LV-GLSRe = LV global longitudinal early diastolic strain rate, LV-GLSRa = LV global longitudinal late diastolic strain rate. Date are expressed as mean ± SD. *p < 0.05. **p < 0.01.

LA-GLSRe significantly correlated with LAVmax (*r* = 0.586, *p* < 0.01), LAV_min_ (*r* = 0.530, *p* < 0.01), LAVp (*r* = 0.564, *p* < 0.01), LAAEF (*r* = 0.340, *p* < 0.05), LA total EF (*r* = 0.256, *p* < 0.05) and LVEF (*r* = –0.477, *p* < 0.001). LA-GLSRa had significant correlations with the following echocardiographic variables: LAV_max_ (*r* = 0.604, *p* < 0.01), LAV_min_ (*r* = 0.615, *p* < 0.01), LAV_p_ (*r* = 0.590, *p* < 0.01) and LAPEF (*r* = –0.298, *p* < 0.05).

**Fig. 5.**
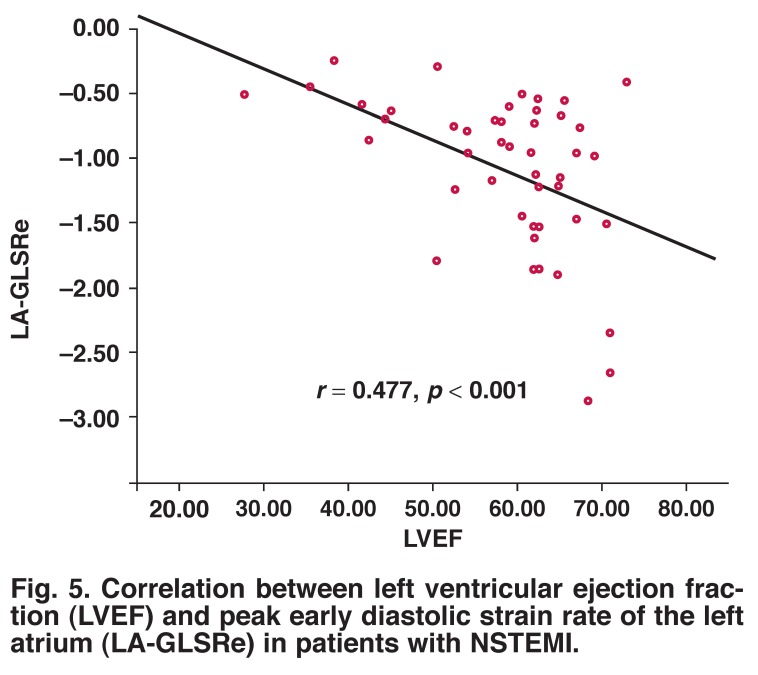
Correlation between left ventricular ejection fraction (LVEF) and peak early diastolic strain rate of the left atrium (LA-GLSRe) in patients with NSTEMI.

LV SR parameters had no significant correlation with the following LA echocardiographic variables: LAV_max_, LAV_min_, LAV_p_, LAPEF, LAAEF and LA total EF. In addition, LVEF was significantly correlated with LA-GLSRs (*r* = –0.334, *p* < 0.05) and LA-GLSRe (*r* = –0.477, *p* < 0.001) (Fig. 5), but not significantly correlated with LA-GLSRa (*r* = 0.339, *p* > 0.05).

**Fig. 6.**
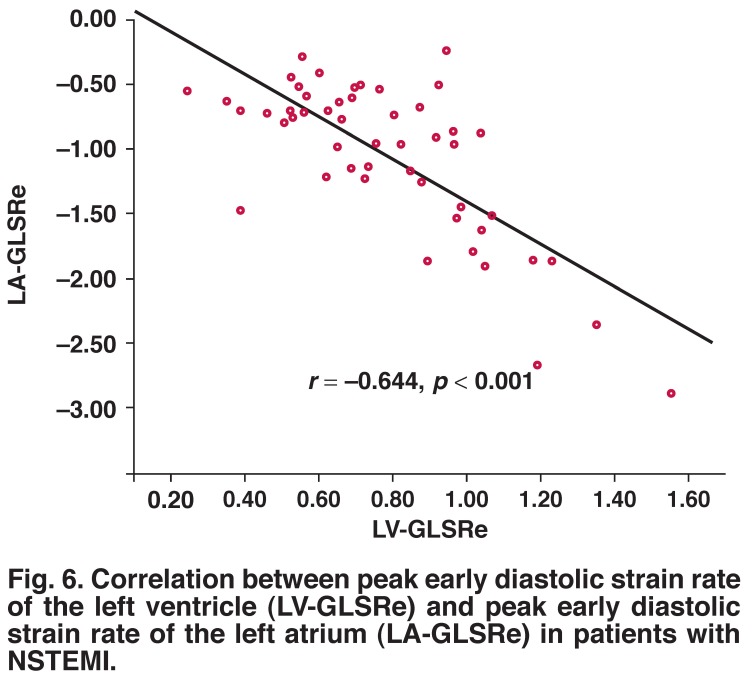
Correlation between peak early diastolic strain rate of the left ventricle (LV-GLSRe) and peak early diastolic strain rate of the left atrium (LA-GLSRe) in patients with NSTEMI.

**Fig. 7.**
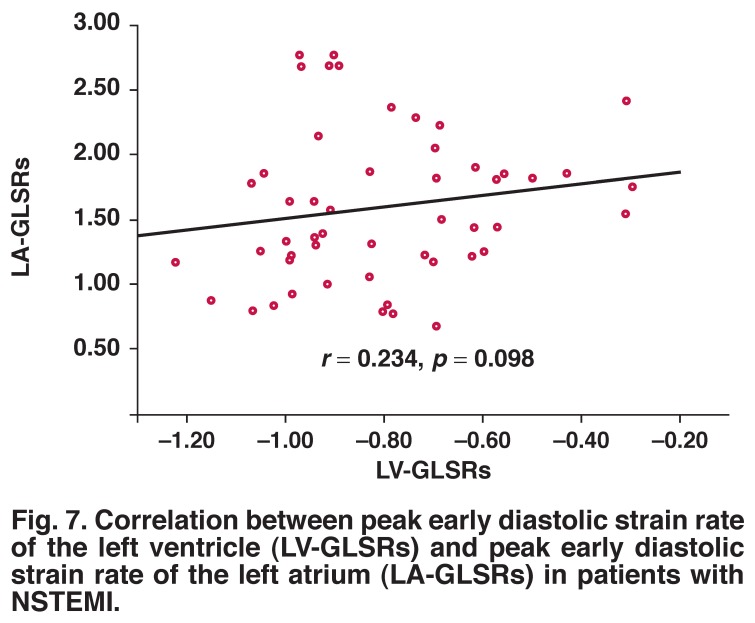
Correlation between peak early diastolic strain rate of the left ventricle (LV-GLSRs) and peak early diastolic strain rate of the left atrium (LA-GLSRs) in patients with NSTEMI.

**Fig. 8.**
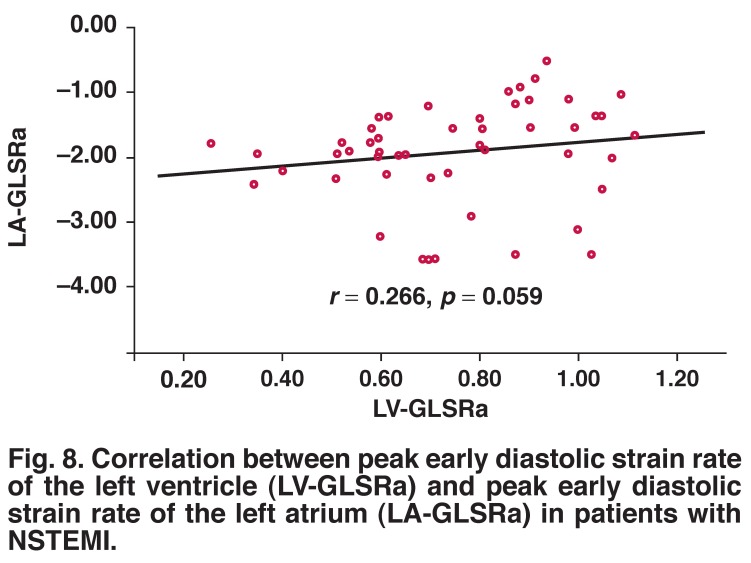
Correlation between peak early diastolic strain rate of the left ventricle (LV-GLSRa) and peak early diastolic strain rate of the left atrium (LA-GLSRa) in patients with NSTEMI.

LA-GLSRe correlated significantly with LV-GLSRe (*r* = –0.644, *p* = 0.001) (Fig. 6). However, both LA-GLSRs and LA-GLSRa showed no such significant correlation with LV-GLSRs (Fig. 7) and LV-GLSRa (Fig. 8), respectively.

## Discussion

After several decades of investigation, current consensus recommendations state that LA function plays an important role in optimising overall cardiac function, and the changes in LA size and function are associated with cardiovascular disease and are risk factors for atrial fibrillation, stroke and death.^12^–^14^ The left atrium serves as a blood reservoir during ventricular systole and a conduit for the passage of blood from the pulmonary veins into the left ventricle during early and middle ventricular diastole, as well as a booster pump increasing LV filling during late diastole.^15^ In subjects with normal diastolic function, the relative contribution of the reservoir, conduit and pump function of the LA to the filling of the LV is approximately 40, 35 and 25%, respectively.^16^

Determined by conventional 2D echocardiography, LA function has been mainly evaluated using LA volumetric parameters and LA emptying fraction, such as LA total EF, LAPEF, and LAAEF, which may be used to evaluate the reservoir, conduit and booster pump components of LA function.^6^,^13^ Parameters that evaluate LA function may have prognostic potential. LA reservoir function may predict the first atrial fibrillation or flutter episode in elderly subjects, and LA systolic force may predict cardiovascular events in a population with a high prevalence of hypertension and diabetes.^17^,^18^

However, all these echocardiographic parameters and others that evaluate LA function are influenced by LV dynamics and geometry and/or rely on measurements that are subjected to error.^19^,^20^ Therefore, new methodologies that can evaluate LA function by analysis of LA myocardial deformation may be of potential clinical interest.

Two strain imaging methods are based on different principles and can potentially give different results. Tissue Doppler imaging (TDI)-derived strain is limited to the measurement of movement parallel to the ultrasound beam. Non-Doppler 2D strain imaging derived from speckle tracking is a newer echocardiographic technique for obtaining SR measurements. The advantage of this method is that it tracks in two dimensions, along the direction of the wall, not along the ultrasound beam, and thus is angle independent, which is a great advantage of non-Doppler 2D strain imaging in comparison to TDI-derived strain data.^2^

Previous studies show that 2D STE with its latest applications such as strain rate imaging may represent promising techniques to better evaluate LA function.^21^ With the use of strain rate imaging, Inaba *et al.* found that SRs corresponded to reservoir function and SRe corresponded to conduit function, while SRa corresponded to booster pump function.^22^

In patients with AMI, left ventricular stroke volume is relatively maintained despite the impairment of left ventricular function caused by myocardial ischaemia and necrosis. With increased stiffness or reduced compliance of the LV, LA pressure rises to maintain adequate LV filling, and the increased atrial wall tension leads to chamber dilatation and stretch of the atrial myocardium.^23^ Therefore, the left atrium works harder and transports more blood to the left ventricle during left ventricular diastole. This function of the left atrium can be attributed to the Frank-Starling mechanism. LA pump function augmentation is therefore due to the increased left atrial volume before active atrial emptying, but not to the increased contractility of the left atrium.^24^

In our study protocol, patients with NSTEMI showed increased LA volumes (LAV_max_, LAV_min_ and LA_p_). Moreover, indices of LA reservoir function (LA total EF) and LA conduit function (LAPEF) were significantly impaired and compared with healthy controls, but LA booster function (LAAEF) seemed to be unchanged in both normal subjects and patients (Table 2).

In accordance with the conventional echocardiographic parameters mentioned above, we found LA reservoir function assessed by SR imaging (LA-GLSRs) and LA conduit function assessed by SR imaging (LA-GLSRe) were significantly reduced in patients with NSTEMI (Table 4), but LA booster function assessed by SR imaging (LA-GLSRa) showed no significant difference. This may be explained by when the LA is well stretched longitudinally, and consequently a high LA positive peak is present, the LV then relaxes rapidly, generating a high E wave, as blood rushes into the LV, generating a high passive LA emptying fraction. Therefore, LA-GLSRs and/or LA-GLSRe have significant correlations with LV diastolic function, which are impaired in patients with NSTEMI.

In our study protocol, a good correlation was found between LA global strain rate and LA functional parameters (Table 5). The present study extends previous results and describes changes in LA function after AMI, combining LA volumes, LA emptying fraction, and LA strain in patients with NSTEMI. The results show that speckle tracking-derived strain rate is a promising technique to assess LA function as well as LA volumes and LA emptying fraction.

Global strain is a relatively new parameter for assessment of LV function^25^ and tends to predict the infarct mass better than established indices of global function such as LVEF and WMSI. LVEF can be regarded as the sum of all LV systolic deformation.

In Wakami *et al.*’s study, peak LA strain rate during LV systole, which corresponds to our measured LA-GLSRs, correlated inversely with LV end-diastolic pressure and LV end-systolic volume and positively with LVEF.^26^ In a recent study by Vartdal *et al.*, global strain measured by TDI immediately after PCI was found to be superior to LVEF for predicting final infarct mass in patients with acute MI.^27^ Comparing with tagged magnetic resonance imaging (the current ‘gold standard’ for deformation analysis), STE measurements correlated well with data obtained by magnetic resonance imaging, both in normal myocardial segments and infarcted areas (*r* = 0.87, *p* < 0.001).^28^

The findings of our present study are in accordance with previous studies. There was significant correlation between LVEF and global LA-GLSRs (*r* = –0.334, *p* < 0.05) or LA-GLSRe (*r* = –0.477, *p* < 0.001). In particular, LA-GLSRe was strongly correlated with LV-GLSRe (*r* = –0.644, *p* = 0.001), while LA-GLSRs and LA-GLSRa were not significantly correlated with LV strain rate parameters (LV-GLSRs and LV-GLSRa). These findings support the idea that LA-GLSRe can serve as an important new marker of LA and LV function in the acute MI.

Therefore, speckle tracking echocardiography was found to be a feasible and reproducible method to assess LA longitudinal strain in healthy subjects and patients with NSTEMI. The reproducibility of measurements was good, with lower variability of intra- and inter-observer. In particular, we found LA-GLSRe was significantly correlated with both LA 2D Doppler echocardiographic parameters and LV contractile function, and could be an optimal parameter of 2D STE in assessing the degree of impairment of heart function in patients with NSTEMI. These data suggest that speckle tracking echocardiography may be considered a promising tool to explore LA myocardial deformation dynamics.

## Study limitations

A number of obvious limitations of our study should be noted. First, the 2D STE analysis software that was originally designed for the left ventricle was applied to the left atrium in our study. Second, echocardiography in this study was not performed in the emergency room but on arrival at the coronary care unit or one to three days later. Third, the relatively small number of patients eligible for analysis in the present study may render it difficult to generalise the results and apply them to other patient populations. Further larger, prospective studies are required to determine the cost effectiveness of this new technique to evaluate LA function in NSTEMI patients. Lastly, this was a cross-sectional study, and therefore no clinical outcomes were examined.

## Conclusions

Our study demonstrated that two-dimensional speckle tracking echocardiography represented a non-invasive, relatively simple and reproducible technique to assess left atrial myocardial function in patients with NSTEMI. Considering the limitations of classical indices of LA function, speckle tracking is easy to operate and has the advantage of being angle independent and less affected by reverberations. The reservoir and conduit function of the left atrium were impaired in these patients, compared with age-matched healthy controls. Importantly, LA-GLSRe was significantly correlated with both LA 2D Doppler echocardiographic parameters and LV contractile function and could be suggested as a better indicator to evaluate LA function as a preferred parameter of STE.
